# Optical genome mapping as a diagnostic tool for unsolved balanced translocations in couples with adverse pregnancy outcomes: a case series

**DOI:** 10.1186/s40001-025-03814-7

**Published:** 2026-01-08

**Authors:** Xiaohuan Zhang, Yingnan Liao, Li Cao, He Lin, Xuemei Wu, Ling Liao, Chang Tan, Shi Ma, Guo Huang

**Affiliations:** Genetic Diseases Key Laboratory of Sichuan Province, Department of Medical Genetics, Department of Laboratory Medicine, Sichuan Academy of Medical Sciences & Sichuan Provincial People’s Hospital, School of Medicine, University of Electronic Science and Technology of China, Chengdu, China

**Keywords:** Optical genome mapping, Complex chromosomal rearrangement, Cryptic translocation, Karyotyping, Trio-whole exome sequencing

## Abstract

**Objective:**

Cryptic balanced translocations are a challenging diagnostic dilemma in conventional cytogenetics. This study aimed to evaluate the utility of optical genome mapping (OGM), an emerging technology for detecting structural variations, in resolving such cases among couples with adverse pregnancy outcomes.

**Method:**

We performed a retrospective analysis using OGM on three unsolved cases following karyotyping and trio-whole exome sequencing (trio-WES). The cohort included two couples with adverse pregnancy outcomes and a 46,XX SRY+ male.

**Results:**

In all three cases where a translocation was clinically suspected but cytogenetically elusive, OGM successfully identified precise cryptic balanced translocations. Crucially, it defined the genomic breakpoints at high resolution. In the case of the 46,XX SRY+ male, OGM identified a cryptic translocation of Yp11.2 into Xp22.3 and the paracentric inversion inv(Y)(p11.2p11.2), explaining the sex reversal phenotype.

**Conclusion:**

This study shows that OGM is a valuable adjunctive diagnostic method for detecting cryptic translocations. By providing a molecular diagnosis, it enables definitive reproductive risk assessment and personalized genetic counseling for carrier couples.

**Supplementary Information:**

The online version contains supplementary material available at 10.1186/s40001-025-03814-7.

## Introduction

Chromosomal translocations, which involve the exchange of genetic material between non-homologous chromosomes, represent a major source of structural variation in the human genome. These rearrangements arise from genetic, physical, or chemical factors and can introduce considerable nucleotide-level diversity [1–4]. Although carriers of balanced translocations are often phenotypically normal, they face significantly elevated risks of recurrent miscarriage, infertility, and abnormal pregnancy outcomes due to the generation of unbalanced chromosomal complements in their offspring [5]. Despite these clinical implications, such cases—along with their associated phenotypic heterogeneity—remain underreported, leaving the underlying pathogenic mechanisms poorly understood.

Current standard-of-care (SOC) diagnostic methods for detecting translocations include karyotyping, chromosome microarray (CMA), fluorescence in situ hybridization (FISH), and next-generation sequencing (NGS) [6, 7]. However, cryptic translocations—characterized by subtle or telomeric chromosomal aberrations—often evade detection by these conventional approaches. Karyotyping, while useful for identifying aneuploidy and large rearrangements, is limited by a resolution of 5–10 Mb. CMA improves upon this resolution for detecting copy-number variants but cannot identify balanced translocations. Although FISH offers cellular-level visualization of specific loci, it requires prior knowledge of the target region and the design of locus-specific probes, restricting its utility in unbiased screening [8]. While low-coverage whole-genome sequencing and long-read sequencing have shown promise in detecting some cryptic rearrangements, both struggle to reliably resolve balanced structural variants, particularly those involving repetitive or complex genomic regions [9–11]. Moreover, long-read sequencing is often hampered by higher assembly error rates and substantial cost [12, 13]. Thus, a rapid, reliable, and comprehensive method for detecting cryptic translocations remains an unmet need in clinical cytogenetics.

OGM has recently emerged as a powerful, sequencing-free technology for detecting a broad spectrum of structural variations, including cryptic translocations [14, 15]. This technique involves labeling high-molecular-weight DNA at specific sequence motifs, linearizing the molecules in nanochannel arrays, and imaging them to generate genome-wide maps with high resolution. By integrating microfluidics, high-resolution microscopy, and automated image analysis, OGM enables de novo genome assembly and comprehensive variant detection from a single assay [16, 17]. It can detect diverse translocations within a single assay enabling the unbiased SV profile, which is advantageous in hematological malignancies [18], solid tumors [19, 20], and prenatal conditions [14].

The present work aimed to evaluate the performance of OGM in detecting cryptic translocations in three Chinese families with a history of unexplained adverse pregnancy outcomes. We further assessed its potential to enhance routine clinical cytogenetics and facilitate accurate reproductive counseling.

## Materials and methods

### Participants and ethical approval

The study adhered to the principles outlined in the Declaration of Helsinki. Approval for the research protocol was obtained from the Ethics Committee of Sichuan Academy of Medical Sciences & Sichuan Provincial People’s Hospital. Prior to participation, all subjects provided written informed consent, specifically covering the use of abortus tissue in research and the publication of anonymized findings.

### G-banding analysis

Standard protocols were followed for routine karyotyping of peripheral blood lymphocytes. Chromosomal abnormalities were carefully examined and analyzed in accordance with the International System for Human Cytogenomic Nomenclature (ISCN, 2024) using a high resolution of 400 bands.

### Trio-whole exome sequencing

Trio-WES was conducted using the Human Exome ProbesP039-Exome kit from MyGenostics in Beijing, China. This was performed on the IlluminaNovaSeq5000 platform. The sequencing achieved a mean exome coverage of over 95% (> 10 × coverage; mean depth of over 100x). To ensure the accuracy and reliability of the data, the reads were subjected to rigorous quality control measures before being mapped to the human reference genome (UCSChg38). Detected variations including short indels and SNVs were carefully annotated and ranked using the VaRank software. To comprehensively interrogate copy number variations, we employed a dual-caller approach to enhance detection sensitivity and specificity. Copy Number Variations (CNVs) were called independently using XHMM. Our analysis pipeline was validated for the detection of intragenic and multi-exonic CNVs, with a size threshold of 1 kb. Called CNVs were filtered based on the following quality control (QC) metrics: Q30 > 85%, 40% < Mean GC per Read < 55%, IQR ≤ 45.

### Optical genome mapping (OGM)

Ultra-high molecular-weight (UHMW) DNA was extracted from peripheral blood using the Bionano Prep SP Frozen Human Blood DNA Isolation Protocol. Subsequently, the Direct Label and Stain (DLS) kit was used to label the DNA according to the manufacturer’s instructions. The specific 6-base pair sequence motif (CTTAAG) was fluorescently labeled. The labeled genomic DNA molecules were then loaded onto a Saphyr chip and run on a Saphyr instrument. Data analysis, including de novo assembly and structural variation (SV) calling, was performed using Bionano Solve v3.7, with alignment to the GRCh38 reference genome. OGM Technical Metrics and Quality Control for All Samples (Supplementary Table S1).

## Results

### Detection of cryptic translocations Using OGM

Two families with a history of adverse pregnancy outcomes or fertility were recruited to this study at the Sichuan Provincial People's Hospital. These probands from case1 and case 2 had previously received trio-WES. Trio-WES identified two CNVs of similar fragment sizes on distinct chromosomes in the probands, including duplication and deletion. According to the classification guidelines of the American College of Medical Genetics (ACMG) for CNV, the deletions and duplications were deemed to be the candidate pathogenic CNV. Trio-WES revealed no clinically significant CNVs or genetic variants in their parents, but balanced translocations were highly suspected based on proband’s trio-WES results. Next, the individuals of two cases were identified to have normal karyotypes. Consequently, the etiology of the adverse pregnancy outcome remains undetermined. In this study, OGM detected two cryptic translocations, which may be related to the phenotypes of patients. Detailed information on these cryptic translocations is described in Table 1.

**Table 1:** Summary of case information, Trio-WES, original karyotypes and OGM results in Case1/2

Sample	Age (years); sex	Clinical data	Trio-WES	Karyotyping	OGM
1P	5, male	hydrocephalus, developmental delay, ptosis, hypospadias, penile curvature, bilateral non-palpable testes, left-hand polydactyly, clubfoot deformity, patent ductus arteriosus, and aortic regurgitation	GRCh38:chr4:174,491,936–190,027,224del GRCh38:chr16:73,441,533–90,075,935dup	46, XY	ogm [GRCh38] der(4)t(4;16)(q34.1;q22.3)(174,413,783–174,428,016;73,375,840–73,383,124)
1F	28, male	N	N	46, XY	ogm[GRCh38] t(4;16)(q34.1;q22.3)(174,413,783–174,428,016;73,375,840–73,383,124)
1 M	29, female	N	N	46, XX	N
2P (abortive tissue)	0	Nuchal translucency thickness of 6.1 mm, lymphatic cyst	GRCh38:chr9:14806-8636844del GRCh38:chr11:122,526,757–134,257,553dup	ND	ogm[GRCh38] der(9)t(9;11)(p24.1;q24.1)(8,856,403–8,869,328; 122,364,368–122,371,304)
2F	31, male	N	N	46, XY	N
2 M	30, female	N	N	46, XX	ogm[GRCh38] t(9;11)(p24.1;q24.1)(8,856,403–8,869,328; 122,364,368–122,371,304)

*ND* Not done, *N* Normal

### A cryptic translocation in case 1

**Fig. 1:**
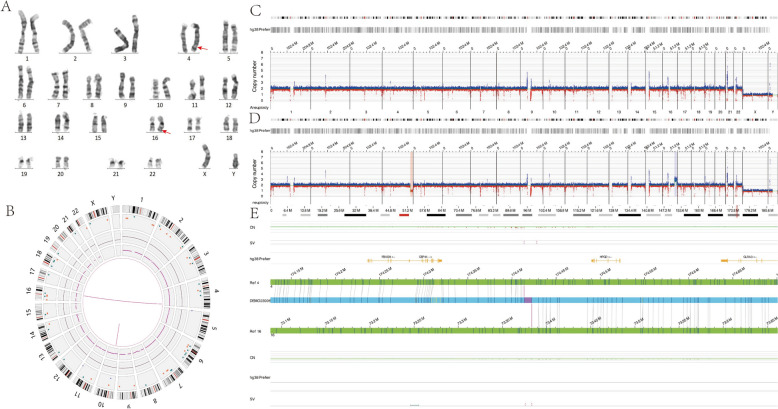
Genome-wide visualisation of OGM data and example of a cryptic balanced translocation in Case 1. **A** Karyotype of the proband's father revealing a normal result. **B** Circos plot from the father’s OGM data indicating a cryptic balanced translocation between chromosomes 4 and 16 (connected by a pink line). **C** The father’s OGM CNV profile showing normal copy number for chromosomes 4 and 16. **D** The proband’s OGM CNV profile showing a microdeletion on 4q34.1 and a microduplication on 16q22.3. **E** OGM results detailing the cryptic balanced translocation between chromosomes 4 and 16 in the father

The proband was a 5-year-old male. Prenatal ultrasound had indicated ventriculomegaly with hydrocephalus, though no specialized treatment was provided. After birth, the child was diagnosed with hydrocephalus, developmental delay, ptosis, hypospadias, penile curvature, bilateral non-palpable testes, left-hand polydactyly, clubfoot deformity, patent ductus arteriosus, and aortic regurgitation. Following clinical evaluation, trio-WES was performed to identify the potential causative genetic causes in the family. While no pathogenic single-nucleotide variants were detected,a 15.535 Mb deletion on chromosome 4 (NC_000004.12:g.174491936_190027224del) and 16.634 Mb duplication of chromosome 16 (NC_000016.10:g.73441533_90075935dup) were identified in the proband. Genetic counseling suggested a cryptic unbalanced translocation between chromosomes 4 and 16, though initial cytogenetic confirmation was lacking. Parental peripheral blood karyotyping showed normal results (Fig. 1A).

To further investigate the suspected cryptic translocation and map the breakpoints precisely, OGM was performed on family members. OGM identified a paternal cryptic balanced translocation, designated as ogm[GRCh38] der(4)t(4;16)(q34.1;q22.3) (174,413,783–174,428,016; 73,375,840–73,383,124) (Fig. 1B). The OGM CNV profile of the father was normal (Fig. 1C), while the proband’s profile showed a microdeletion at 4q34.1 and a microduplication at 16q22.3, consistent with inheritance of the rearranged segments from the father (Fig. 1D). These OGM findings were concordant with the trio-WES CNV results. Furthermore, OGM refined the breakpoints to chr4:174,413,783–174,428,016 and chr16:73,375,840–73,383,124 (Fig. 1E).

### A cryptic translocation in case 2

A 30-year-old pregnant woman was referred for genetic counseling due to an adverse prenatal finding. Fetal ultrasound shows a nuchal translucency (NT) thickness of 6.1 mm, with fluid accumulation in the head and neck region suggestive of a lymphatic cyst. After pregnancy termination, trio-WES was performed on the abortion tissue, which revealed an 8.62 Mb deletion on chromosome 9 (NC_000009.12:g.14806_8636844del) and an 11.73 Mb duplication on chromosome 11 (NC_000011.10:g.122526757_134257553dup). According to the classification guidelines of ACMG for CNV, the 9p24.3-p24.1 deletion and 11q24.1-q25 duplication were both classified as likely pathogenic.

**Fig. 2:**
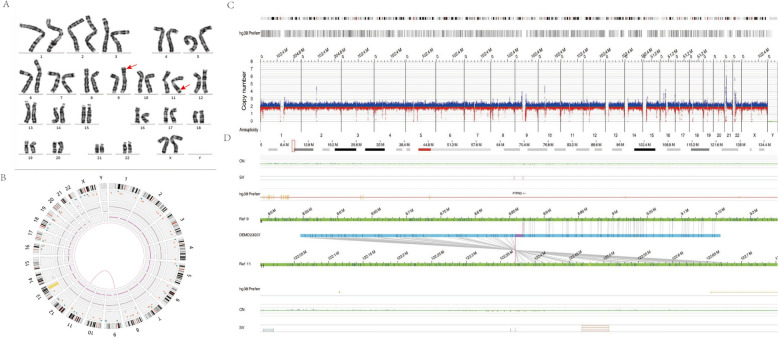
Genome-wide visualisation of OGM data and example of a crypticbalanced translocation in Case 2. **A** Karyotype of the proband's mother showing a normal result. **B** Circos plot from the mother's OGM data indicating a cryptic balanced translocation between chromosomes 9 and 11 (connected by a pink line). **C** The mother's OGM CNV profile showing normal copy number for chromosomes 9 and 11. **D** OGM results detailing the cryptic balanced translocation between chromosomes 9 and 11 in the mother

Genetic counseling suggested that the proband’s CNV pattern resulted from a cryptic unbalanced translocation involving chromosomes 9 and 11. No initial cytogenetic evidence was available. Parental peripheral blood karyotyping was normal (Fig. 2A). To further investigate the suspected translocation and precisely map the breakpoints, optical genome mapping (OGM) was performed on the couple. OGM analysis successfully identified a maternal balanced translocation, designated as ogm[GRCh38] der(9)t(9;11)(p24.1;q24.1) (8,856,403–8,869,328; 122,364,368–122,371,304) (Fig. 2B). The mother’s OGM CNV profile was normal (Fig. 2C), consistent with a balanced rearrangement. The breakpoints were precisely mapped to chr9:8,856,403–8,869,328 and chr11:122,364,328–122,371,304 (Fig. 2D).

### A cryptic translocation in case 3

**Fig. 3:**
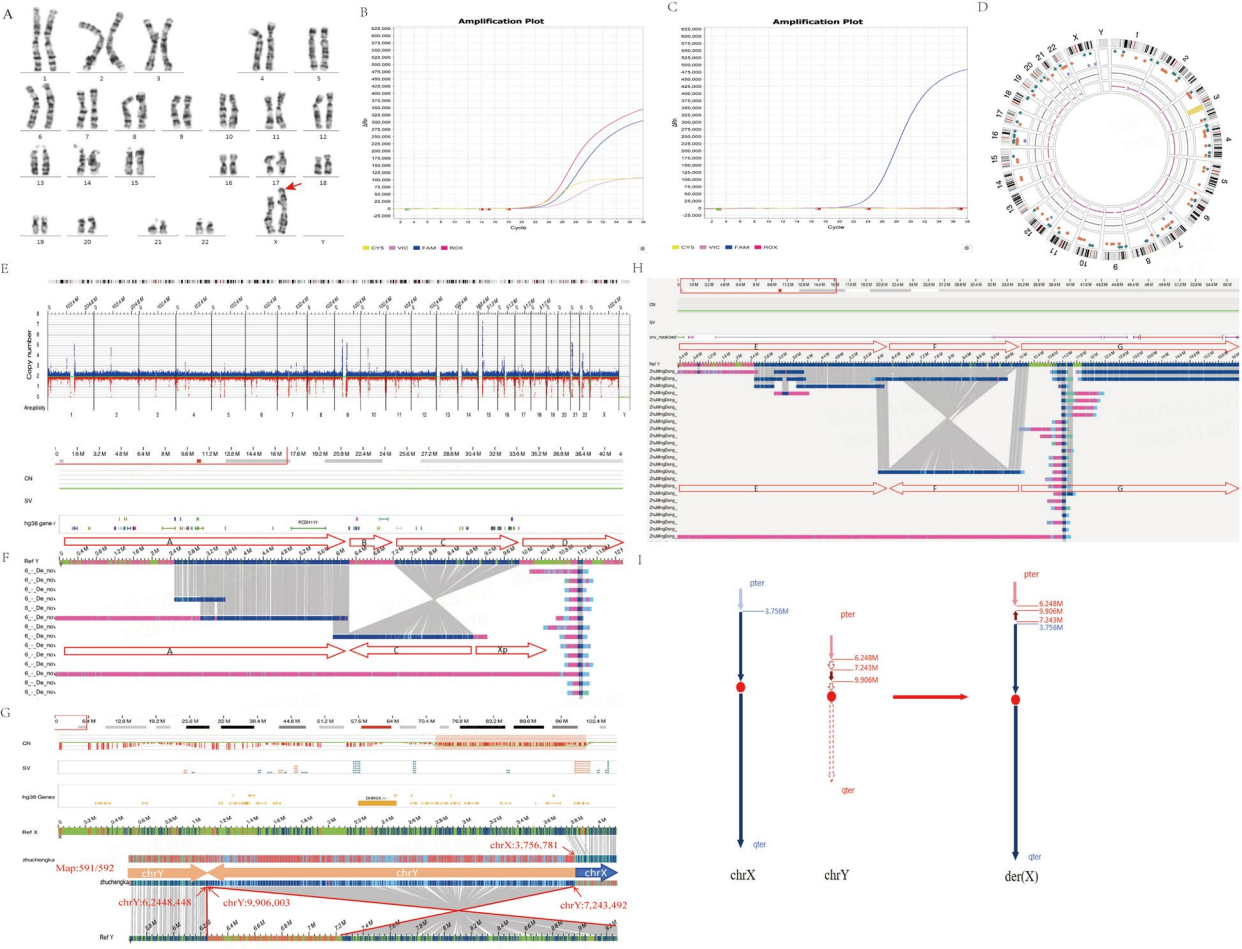
OGM reveals a cryptic translocation in Case 3. **A** Conventional karyotype of the proband showing a 46,XX. **B-C** Quantitative PCR detection of the SRY gene (B: control; C: proband). **D** Circos plot of genome-wide OGM showing no evidence of Y-derived chromatin. **E** OGM-based CNV profile showing no evidence of Y-derived chromatin. **F** OGM SV analysis identifying a complex rearrangement between X and Y chromosomes, including deletions, translocations, and an inversion. **G** Genome map view showing the cryptic translocation of Yp11.2 into Xp22.3 and the paracentric inversion. **H** OGM SV analysis identifying a benign polymorphic inversion in segment F of the father’s Y chromosome ogm[GRCh38] inv(Y)(p11.2p11.2)(6,248,448_9,906,003). **I** Schematic of the proband's derivative X chromosome

The proband was a 21-year-old male presenting with abnormal sex hormone levels, azoospermia, and varicocele. Laboratory tests indicated increased levels of progesterone (1.12 nmol/L; normal, 0.159–0.474), luteinizing hormone (13 mIU/mL; normal, 1.7–8.6), prolactin (797 mIU/L; normal, 86–324), and follicle-stimulating hormone (15.6 mIU/mL; normal, 1.5–12.4). Blood estrogen (132 pmol/L; normal, 41.4–159) and testosterone (13 nmol/L; normal, 6.52–30.6) levels were within normal. Peripheral blood karyotype analysis revealed a 46, XX (Fig. 3A). Nevertheless, SRY gene was clearly defined in the proband by Quantitative PCR (Fig. 3B, C). Genetic counseling raised strong suspicion of a cryptic translocation event between chromosome X and Y. The patient articulated a desire or procreation and intended to further confirm the genetic modification. OGM was performed on all family members. OGM SV analysis identified a complex rearrangement involving the short arms of the X and Y chromosomes in the proband. This included: deletion of segments B and D and part of the long arm on the Y chromosome; translocation and fusion between segments A and C; and an inversion of segment C followed by its fusion with the Xp22.33 region (Fig. 3D–G). OGM SV analysis detected a benign polymorphic inversion in segment B of the father's Y chromosome(ogm[GRCh38] inv(Y)(p11.2p11.2)(6,248,448_9,906,003)), structurally similar to the inversion in segment C observed in the proband (Fig. 3H). The final interpretation was a cryptic translocation defined as ogm[GRCh38] der(X)(Ypter_Yp11.2)(pter_6,248,448)::(Yp11.2_Yp11.2)(9,906,003_7,243,492)::(Xp22.33_Xqter)(3,756,781_qter)No maternal chromosomal CNVs or translocations were identified. Details are shown in Table 2 and Fig. 3.

**Table 2:** Summary of case information, karyotyping, *SRY* gene status, and OGM results for Case 3

Sample	Age (years); sex	Clinical data	*SRY* gene	Karyotyping	OGM
3P	21, male	gender-reversed male, abnormal sex hormone levels, azoospermia, varicocele	*SRY*gene(+) *AZF*gene(−)	46, XX	ogm[GRCh38] der(X)(Ypter_Yp11.2)(pter_6,248,448)::(Yp11.2_Yp11.2)(9,906,003_7,243,492)::(Xp22.33_Xqter)(3,756,781_qter)
3F	43, male	N	ND	46, XY	ogm[GRCh38] inv(Y)(p11.2p11.2)(6,248,448_9,906,003)
3 M	42, female	N	ND	46, XX	N

*ND* Not done, *N* Normal

## Discussion

This study demonstrates that OGM holds significant value in identifying cryptic balanced translocations that are difficult to detect using traditional cytogenetic methods. In all three families with unexplained adverse reproductive outcomes, OGM successfully identified the chromosomal rearrangements which had remained undetected by conventional karyotyping and WES, providing precise molecular diagnoses for these families.

Our findings are consistent with several recent reports, supporting the application prospects of OGM in clinical cytogenetics [21–23]. Compared with traditional karyotyping, OGM achieves comprehensive, genome-wide screening for structural variations with significantly higher resolution [24, 25]. In Cases 1 and 2, the fragment lengths involved in the balanced translocation between chromosomes 4 and 16 and the balanced translocation between chromosomes 9 and 11 both exceed 5 Mb. However, they went undetected by G-banding analysis due to the similar size and banding pattern of the exchanged segments. By directly visualizing the DNA molecular structure, OGM not only confirmed these rearrangement events but also enabled precise breakpoint mapping [26, 27].

Case 3 presented a more complex sex chromosome rearrangement. The 46,XX SRY-positive male phenotype, coupled with the absence of Y chromosome in standard analysis, suggested a cryptic translocation between chromosomes X and Y. OGM analyzed the underlying mechanism: translocation of the Yp11.2 region (containing the *SRY* gene) to Xp22.33, accompanied by a paracentric inversion on Yp11.2. The FISH results (Supplementary Fig. 1) revealed the presence of two X centromeres, a deletion in the Y heterochromatic region, and two signals each on the short-arm and long-arm termini of the sex chromosomes, confirming the suspected OGM result. Additionally, the benign polymorphic inversion found in the Y chromosome F region of the father was structurally similar to the pathogenic inversion in the C region of the proband, providing clues for understanding the susceptibility to rearrangement in this region.

From a clinical translation perspective, OGM results directly altered genetic counseling and management strategies. For families with confirmed parental balanced translocation (e.g., cases 1 and 2), we can provide more accurate recurrence risk assessment and recommend preimplantation genetic testing for structural rearrangement (PGT-SR) as a feasible option for achieving a healthy pregnancy [28]. Based on our experience, we propose a clinical pathway applicable to “unexplained adverse pregnancy outcomes”: when standard testing (SOC) fails to provide diagnostic information, OGM can be considered as a subsequent evaluation method. When OGM identifies a specific rearrangement, targeted prenatal diagnosis or PGT-SR can be provided for family members.

Although OGM shows significant advantages, its technical limitations still need to be carefully considered [29]. The three case series in this study are representative, but the sample size is limited, and insufficient to evaluate the sensitivity/specificity of this technology. We agree that OGM can be used as an early assessment reference when SOC fails to provide information, and this view is supported by large-scale benchmark studies. Concurrently, OGM has limited detection ability for low-proportion chimeras (< 20%) and small fragment variants (< 500 bp) [29]. Inadequate coverage of high-repetitive regions such as centromeres may affect the detection of related rearrangements [30]. Particularly noteworthy is that in Case 3, OGM’s CNV analysis failed to accurately identify Y-chromosomal material, reflecting specific limitations of the current algorithm in sex chromosome analysis—when the Y chromosome retains only the short arm, the software may fail to accurately calculate its copy number changes [31].

Larger multi-center studies are needed to comprehensively evaluate the performance of OGM in different populations and clinical scenarios, and establish corresponding structural variation databases. Nevertheless, this study, through precise molecular diagnosis of three independent families, has confirmed the unique value of OGM in solving complex genetic cases. By integrating OGM into the clinical diagnostic pathway, we can provide more accurate genetic counseling to families experiencing repeated reproductive failures, expand their reproductive options, and ultimately drive clinical cytogenetics towards precision medicine.

## Supplementary Information

Supplementary Material 1.

Supplementary Material 2.

Supplementary Material 3.

## Data Availability

Data supporting our findings are available upon reasonable request by the corresponding author.

## Abbreviations

OGM: Optical genome mapping
SVs: Structural variations
trio-WES: Trio-whole exome sequencing
SOC: Standard-of-care
CMA: Chromosome microarray
FISH: Fluorescence in situ hybridization
NGS: Next-generation sequencing
UHMW: Ultra-high molecular-weight
ACMG: American College of Medical Genetics
QC: Quality control
CNVs: Copy number variations
NT: Nuchal translucency
PGT-SR: Preimplantation genetic testing for structural rearrangement
