# Supplementary material for: Optical genome mapping as a diagnostic tool for unsolved balanced translocations in couples with adverse pregnancy outcomes: a case series
Source: Eur J Med Res. 2026 Jan 8;31:223. doi: 10.1186/s40001-025-03814-7 (PMC12874668; doi:10.1186/s40001-025-03814-7)
Supplement: Supplementary file 1 — Supplementary Material 1. [file 40001_2025_3814_MOESM1_ESM.doc]

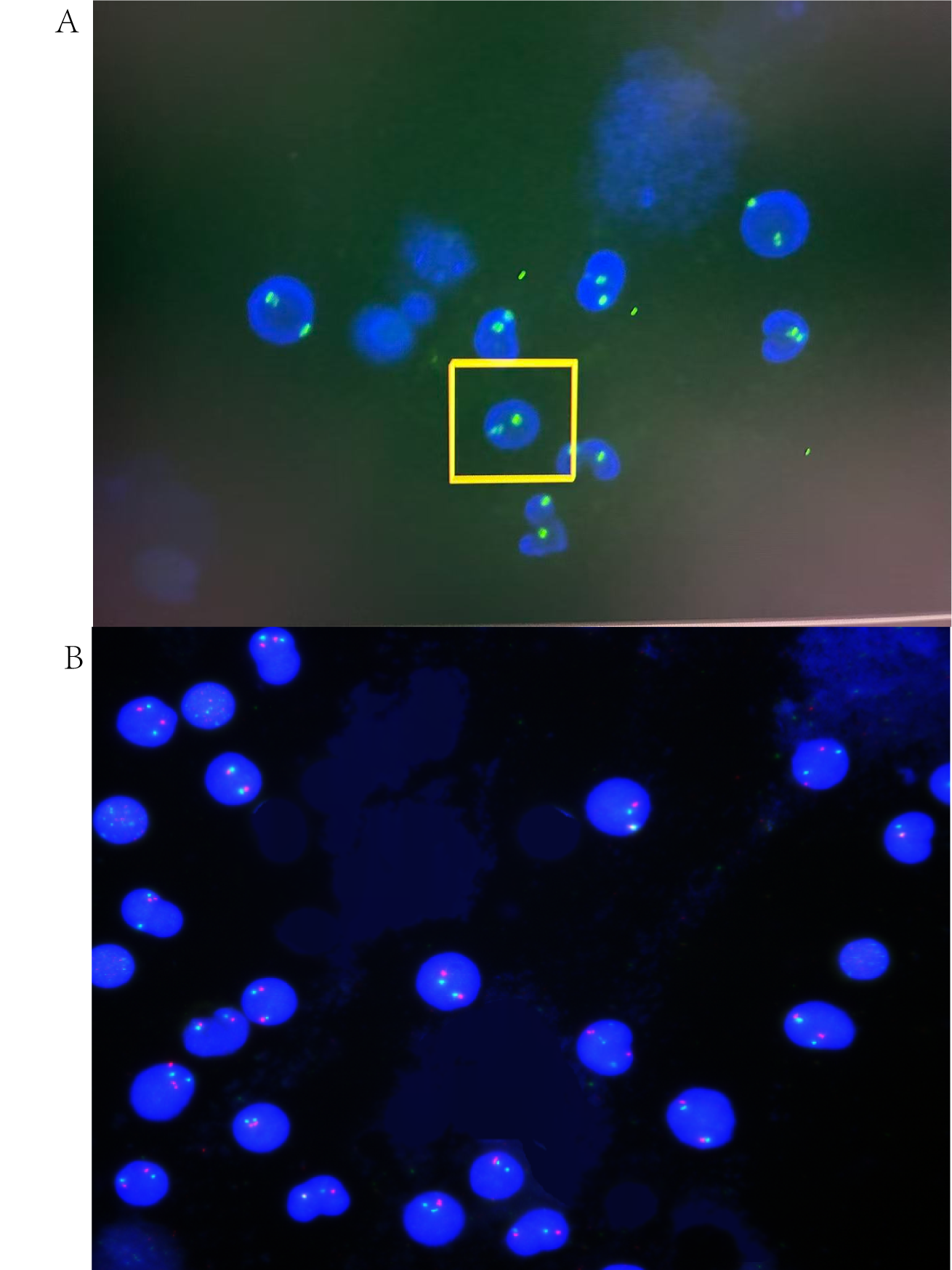


**Supplementary Figure 1. The FISH results of proband in case 3. A** FISH analysis using CSPX(green)/CSPY (red)probes confirmed the presence of two X chromosome centromeres and the absence of the Y heterochromatic region in the subject. **B** FISH analysis using GSP XYqter(green, located at Xp22 and Yp11)/GSP XYpter(red, located at Xq28 and Yq12) probes confirmed the presence of two chromosome ends.
