# Supplementary material for: Optical genome mapping as a diagnostic tool for unsolved balanced translocations in couples with adverse pregnancy outcomes: a case series
Source: Eur J Med Res. 2026 Jan 8;31:223. doi: 10.1186/s40001-025-03814-7 (PMC12874668; doi:10.1186/s40001-025-03814-7)
Supplement: Supplementary file 2 — Supplementary Material 2. [file 40001_2025_3814_MOESM2_ESM.docx]

**Supplementary Table S1** Optical Genome Mapping (OGM) Technical Metrics and Quality Control for All Samples

| Sample ID | Label Density (labels/100kb) | Map Rate (%) | Molecule N50 (kb) | Assembly N50 (Mb) | Effective Enzyme | Pipeline Modules | SV Confidence Filters |
| --- | --- | --- | --- | --- | --- | --- | --- |
| 1P | 16.556 | 72.3 | 325.228 | 773542.289 | DLE-1 | Bionano Solve v3.7 | SVs except translocation with >50% interval  overlap and >80% size overlap are collapsed;  translocation calls within 35kb are collapsed |
| 1F | 16.322 | 76.2 | 354.947 | 762155.321 | DLE-1 | Bionano Solve v3.7 | SVs except translocation with >50% interval  overlap and >80% size overlap are collapsed;  translocation calls within 35kb are collapse |
| 1M | 16.024 | 78.1 | 331.804 | 759000.378 | DLE-1 | Bionano Solve v3.7 | SVs except translocation with >50% interval  overlap and >80% size overlap are collapsed;  translocation calls within 35kb are collapse |
| 2F | 16.242 | 69.1 | 352.407 | 417559.241 | DLE-1 | Bionano Solve v3.7 | SVs except translocation with >50% interval  overlap and >80% size overlap are collapsed;  translocation calls within 35kb are collapse |
| 2M | 16.125 | 73.4 | 279.103 | 377373.229 | DLE-1 | Bionano Solve v3.7 | SVs except translocation with >50% interval  overlap and >80% size overlap are collapsed;  translocation calls within 35kb are collapse |
| 3P | 15.798 | 70.1 | 228.952 | 295165.819 | DLE-1 | Bionano Solve v3.7 | SVs except translocation with >50% interval  overlap and >80% size overlap are collapsed;  translocation calls within 35kb are collapse |
| 3F | 15.816 | 76.9 | 241.524 | 806247.382 | DLE-1 | Bionano Solve v3.7 | SVs except translocation with >50% interval  overlap and >80% size overlap are collapsed;  translocation calls within 35kb are collapse |
| 3M | 15.652 | 71.5 | 289.493 | 361586.708 | DLE-1 | Bionano Solve v3.7 | SVs except translocation with >50% interval  overlap and >80% size overlap are collapsed;  translocation calls within 35kb are collapse |
