# Supplementary material for: Optical genome mapping as a diagnostic tool for unsolved balanced translocations in couples with adverse pregnancy outcomes: a case series
Source: Eur J Med Res. 2026 Jan 8;31:223. doi: 10.1186/s40001-025-03814-7 (PMC12874668; doi:10.1186/s40001-025-03814-7)
Supplement: Supplementary file 3 — Supplementary Material 3. [file 40001_2025_3814_MOESM3_ESM.doc]

**Supplementary Table S2 Gene mapping in OGM SVs breakpoints**

| Sample ID | **OGM** | **Breakpoint 1** | **Gene mapping in breakpoints 1** | **Breakpoint 2** | **Gene**  **mapping in breakpoints 2** | **Breakpoint 3** | **Gene**  **mapping in breakpoints 3** | **Breakpoint 4** | **Gene**  **mapping in breakpoints 4** |
| --- | --- | --- | --- | --- | --- | --- | --- | --- | --- |
| **1F** | ogm[GRCh38] t(4;16)(q34.1;q22.3)(174413783~174428016;73375840~73383124) | chr4:174413783~174428016 | - | chr16:73375840~73383124 | *-* | - | - | - | - |
| **2M** | ogm[GRCh38] t(9 ;11)(p24.1;q24.1)(8,856,403~8,869,328; 122,364,368~122,371,304) | chr9:8,856,403~8,869,328 | *PTPRD* | chr11:122,364,328~122,371,304 | - | - | - | - | - |
| **3P** | ogm[GRCh38] der(X)(Ypter_Yp11.2)(pter_6,248,448)::(Yp11.2_Yp11.2)(9,906,003_7,243,492)::(Xp22.33_Xqter)(3,756,781_qter) | chrX:3,756,781 | - | chrY:7,243,492 | - | chrY:6,248,448 | *TSPY2* | chrY:9,906,003 | - |
